# Extrahepatic Disease in Hepatocellular Carcinoma: Do We Always Need Whole-Body CT or Is Liver MRI Sufficient? A Subanalysis of the SORAMIC Trial

**DOI:** 10.3390/biomedicines10051156

**Published:** 2022-05-18

**Authors:** Thomas Geyer, Philipp M. Kazmierczak, Ingo G. Steffen, Peter Malfertheiner, Bora Peynircioglu, Christian Loewe, Otto van Delden, Vincent Vandecaveye, Bernhard Gebauer, Maciej Pech, Christian Sengel, Irene Bargellini, Roberto Iezzi, Alberto Benito, Christoph J. Zech, Antonio Gasbarrini, Kerstin Schütte, Jens Ricke, Max Seidensticker

**Affiliations:** 1 Department of Radiology, University Hospital, LMU Munich, Marchioninistr. 15, 81377 Munich, Germany; philipp.kazmierczak@med.uni-muenchen.de (P.M.K.); ingo.steffen@charite.de (I.G.S.); peter.malfertheiner@med.uni-muenchen.de (P.M.); jens.ricke@med.uni-muenchen.de (J.R.); max.seidensticker@med.uni-muenchen.de (M.S.); 2 Department of Medicine II, University Hospital, LMU Munich, Marchioninistr. 15, 81377 Munich, Germany; 3 Department of Radiology, School of Medicine, Hacettepe University, Sihhiye Campus, Ankara 06100, Turkey; borapeynir@gmail.com; 4 Section of Cardiovascular and Interventional Radiology, Department of Bioimaging and Image-Guided Therapy, Medical University of Vienna, 1090 Vienna, Austria; christian.loewe@meduniwien.ac.at; 5 Department of Radiology and Nuclear Medicine, Academic Medical Center, University of Amsterdam, 1105 Amsterdam, The Netherlands; o.m.vandelden@amc.uva.nl; 6 Department of Radiology, University Hospitals Leuven, 3000 Leuven, Belgium; vincent.vandecaveye@uzleuven.be; 7 Department of Radiology, Charité-Universitätsmedizin Berlin, 10117 Berlin, Germany; bernhard.gebauer@charite.de; 8 Department of Radiology and Nuclear Medicine, University of Magdeburg, 39106 Magdeburg, Germany; maciej.pech@med.ovgu.de; 9 Radiologie Interventionnelle Vasculaire et Percutanée, CHU de Grenoble, 38043 Grenoble, France; csengel@chu-grenoble.fr; 10 Division of Interventional Radiology, Azienda Ospedaliero Universitaria Pisana, 56126 Pisa, Italy; irenebargellini@hotmail.com; 11 Dipartimento di Diagnostica per Immagini, Radioterapia Oncologica ed Ematologia, Fondazione Policlinico Universitario A. Gemelli IRCCS, UOC di Radiologia, 00168 Rome, Italy; roberto.iezzi.md@gmail.com; 12 Abdominal Radiology Unit, Department of Radiology, Clínica Universidad de Navarra, Universidad de Navarra, 31008 Pamplona, Spain; albenitob@unav.es; 13 Radiology and Nuclear Medicine, University Hospital Basel, University of Basel, 4001 Basel, Switzerland; christoph.zech@usb.ch; 14 Fondazione Policlinico Gemelli IRCCS, Università’ Cattolica del Sacro Cuore, 00168 Rome, Italy; antonio.gasbarrini@policlinicogemelli.it; 15 Department of Gastroenterology, Hepatology and Infectious Diseases, Otto-Von-Guericke University, 39106 Magdeburg, Germany; 16 Department of Internal Medicine and Gastroenterology, Niels-Stensen-Kliniken Marienhospital, 49074 Osnabrueck, Germany

**Keywords:** hepatocellular carcinoma, extrahepatic disease, liver MRI, patient management, therapeutic decision-making

## Abstract

**Background:** To investigate whole-body contrast-enhanced CT and hepatobiliary contrast liver MRI for the detection of extrahepatic disease (EHD) in hepatocellular carcinoma (HCC) and to quantify the impact of EHD on therapy decision. **Methods:** In this post-hoc analysis of the prospective phase II open-label, multicenter, randomized controlled SORAMIC trial, two blinded readers independently analyzed the whole-body contrast-enhanced CT and gadoxetic acid-enhanced liver MRI data sets of 538 HCC patients. EHD (defined as tumor manifestation outside the liver) detection rates of the two imaging modalities were compared using multiparametric statistical tests. In addition, the most appropriate treatment recommendation was determined by a truth panel. **Results:** EHD was detected significantly more frequently in patients with portal vein infiltration (21% vs. 10%, *p* < 0.001), macrovascular infiltration (22% vs. 9%, *p* < 0.001), and bilobar liver involvement (18% vs. 9%, *p* = 0.006). Further on, the maximum lesion diameter in patients with EHD was significantly higher (8.2 cm vs. 5.8 cm, *p* = 0.002). CT detected EHD in significantly more patients compared to MRI in both reader groups (*p* < 0.001). Higher detection rates of EHD in CT led to a change in management only in one patient since EHD was predominantly present in patients with locally advanced HCC, in whom palliative treatment is the standard of care. **Conclusions:** Whole-body contrast-enhanced CT shows significantly higher EHD detection rates compared to hepatobiliary contrast liver MRI. However, the higher detection rate did not yield a significant impact on patient management in advanced HCC.

## 1. Introduction

Hepatocellular carcinoma (HCC) is the most common malignant primary liver tumor with a heterogeneous antigenic constellation and varying microvascular density [1,2]. Approximately 10 to 15% of HCC patients present with extrahepatic disease (EHD) at initial diagnosis [3,4]. At the progressive disease stage after locoregional therapy, metastases can even be detected in up to 25% [4,5,6]. It was shown that the prevalence of EHD yields a significant impact on prognosis and treatment decision [7,8,9].

In contrast to other solid malignant tumors, the diagnosis of HCC may be made based solely on contrast-enhanced imaging in the majority of cases, without the need for additional tissue sampling [10]. For tumor detection and staging at initial diagnosis, the current guidelines by the American Association for the Study of Liver Diseases (AASLD) and the European Association for the Study of the Liver (EASL), recommend multiphasic contrast-enhanced imaging: ultrasound and computed tomography (CT) with extracellular contrast agents or magnetic resonance imaging (MRI) with combined extra- and intracellular (hepatobiliary) contrast agents, respectively [11]. However, MRI with a hepatobiliary contrast agent remains the imaging reference standard for the detection and differential diagnosis of HCC and other focal liver lesions, as it demonstrates a significantly higher sensitivity and specificity compared to contrast-enhanced ultrasound and CT, particularly for smaller lesions with a diameter <1 cm [10,11,12,13,14].

In addition to dedicated liver MRI, the European Society for Medical Oncology (ESMO) clinical practice guidelines for the diagnosis, treatment, and follow-up of HCC, recommend CT of the chest, abdomen, and pelvis to rule out EHD [15]. Several studies indicate a benefit of whole-body contrast enhanced CT staging in addition to liver imaging for the detection of EHD [7]. To date, however, it remains to be elucidated if whole-body CT is indicated in every HCC patient or should only be performed in selected HCC patient subsets.

Taking into consideration the high frequency of diagnostic imaging in HCC patients (i.e., for the initial diagnosis and follow-up after therapy), it would be desirable to differentiate patients who benefit from combined liver MRI and whole-body CT from those in whom additional CT does not impact the therapy decision. Hereby, the associated costs and logistical effort of dual imaging could be reduced.

The aim of the present study was to investigate the performance of whole-body contrast-enhanced CT and hepatobiliary contrast liver MRI for the detection of EHD in HCC and to investigate the impact of EHD as detected by CT or MRI on therapy decision. We hypothesized that whole-body CT detects EHD in significantly more patients with HCC compared to liver MRI but that this impacts the therapy decision only in a small subset of patients dependent on the primary stage of liver disease.

## 2. Materials and Methods

The present study is a post-hoc analysis of the prospective phase II open-label, multicenter, randomized controlled SORAMIC trial (SORAfenib in combination with local MICro-therapy guided by gadolinium-EOB-DTPA-enhanced MRI, EudraCT 2009-012576-27, NCT0112 6645) comprising diagnostic, local ablation, and palliative study arms in HCC patients [16,17,18]. The three study arms focused on the performance of Gd-EOB-DTPA (gadoxetic acid: Eovist and Primovist)-enhanced MRI versus contrast-enhanced computed tomography (CT) for stratifying patients to a local ablative or palliative therapy, on the value of radiofrequency ablation (RFA) plus sorafenib versus radiofrequency ablation, plus placebo on time to recurrence in the curative arm, and the impact of Yttrium-90 (Y90) radioembolization combined with sorafenib compared to sorafenib alone on overall survival in the palliative arm. The study was approved by the institutional review boards of all participating centers of the SORAMIC trial and was conducted according to the principles expressed in the Declaration of Helsinki/Edinburgh 2002. Informed consent was obtained from all subjects involved in the study.

### 2.1. Study Population

The SORAMIC trial consisted of a diagnostic, a curative, and a palliative study arm. In the diagnostic study arm, a total of 692 patients were included. All patients successfully screened were part of the diagnostic arm and underwent hepatobiliary contrast-enhanced liver MRI and contrast-enhanced whole-body CT. A total of 154 patients was excluded due to incomplete imaging (missing contrast-enhanced CT, gadoxetic acid-enhanced MRI, or both) or missing MRI sequences. Our post-hoc analysis assessed the data of 538 patients with complete imaging data. Among these patients, EHD was present in 74 patients according to the truth panel (see below).

### 2.2. Imaging

Baseline contrast-enhanced CT of the chest, abdomen, and pelvis and gadoxetic acid-enhanced liver MRI were performed according to a standardized protocol [17]. CT scans included an unenhanced, an arterial phase (15 s delay after bolus tracking in the aorta), and a portal venous phase (50 s delay after injection) of the upper abdomen, followed by a whole-body (chest, abdomen, and pelvis) scan in the delayed phase (120 s delay after injection). Gadoxetic acid-enhanced liver MRI included unenhanced T1-weighted gradient echo (GRE) sequences (2D and 3D). Upon intravenous application of gadoxetic acid (Eovist/Primovist, Bayer Vital GmbH, Leverkusen, Germany; weight-adapted doses: 0.1 mL/kg body weight; injection rate 1.5 mL/s), dynamic T1-weighted GRE 2D sequences were performed in the arterial phase (15 s delay), the portal venous phase (60–70 s delay), and the delayed phase (120 s delay). Furthermore, the MRI protocol included T2-weighted turbo spin-echo 2D sequences with and without fat suppression and non-mandatory diffusion-weighted imaging (DWI). T1-weighted GRE 2D sequences were also acquired in the hepatobiliary phase 20 min after injection.

### 2.3. Clinical Assessment

At the time of study inclusion and at each follow-up, clinical data including Child-Pugh score, Eastern Cooperative Oncology Group (ECOG) performance status, bilirubin, alpha-fetoprotein, albumin, and international normalized ratio platelets were assessed.

### 2.4. Blinded Image Read and Truth Panel Assessment

CT and MRI data sets were read by two blinded and independent reader groups, consisting of one board-certified radiologist (reader group 1 = RG1) and six board-certified radiologists (reader group 2 = RG2), respectively. All scans were evaluated regarding image quality, intrahepatic disease, vascular infiltration, treatment recommendation, and EHD. Each read was performed in two separate sessions. Either MRI or CT images of one patient were read in the first session. After a minimum period of 14 days, the additional images (MRI or CT) were presented to the readers, minimizing potential recall bias.

All scans were evaluated by a truth panel consisting of a hepatologist managing HCC patients for more than 10 years and a radiologist with more than 10 years of experience in abdominal diagnostic imaging and interventional HCC therapy. All truth panel decisions were made by consensus. If no consensus was reached, the scans were evaluated by another radiologist of similar experience. The truth panel analyzed all CT and MRI images from baseline and all follow-ups during the first year and all available clinical information. The most appropriate treatment recommendation, consisting of either curative radiofrequency ablation (RFA), palliative treatment, or neither of these, was determined by majority of votes based on the European Association for the Study of the Liver (EASL) Clinical Practice Guidelines for the management of hepatocellular carcinoma [11].

### 2.5. Statistical Analysis

Statistical analysis was performed using dedicated statistics software (SAS version 9.4, SAS Institute, Cary, NC, USA; R Project version 3.5.1, R Foundation for Statistical Computing). Baseline characteristics were compared according to the presence of EHD (EHDyes/EHDno) using a Pearson’s chi-squared test with Yates’ continuity correction. The maximum lesion diameter (median and inter-quartile range) was calculated using a Wilcoxon rank sum test with continuity correction. Odds ratios were calculated to compare the detection rates of EHD of CT vs. MRI. A McNemar’s chi-squared test was used to compare the treatment recommendations of the reader groups to the truth panel decision. The level of significance was set at alpha = 0.05.

## 3. Results

Between 01/2011 and 04/2016, 538 patients were included in the diagnostic arm of the SORAMIC trial. Baseline characteristics are described in Table 1. The baseline characteristics are displayed according to the presence of EHD (EHDyes/EHDno) assessed by the truth panel. In patients with portal vein infiltration, EHD was detected significantly more frequently (21.3%) compared to patients without portal vein infiltration (10.2%) (*p* < 0.001). Similar findings were detected regarding macrovascular infiltration (PVI plus hepatic vein infiltration) (22.9% vs. 9.2%, *p* < 0.001). Furthermore, EHD occurred significantly more often in patients with bilobar liver involvement (18%) compared to patients with unilobar involvement (9%) (*p* = 0.006). Higher maximum hepatic lesion diameters were found in patients with EHD (median maximum diameter 8.2 cm and 5.8 cm in patients without EHD, *p* = 0.002). Moreover, EHD was detected in only 2 out of 116 patients (1.7%) within the Up-to-7-criteria compared to 67 out of 383 patients (17.5%) without the Up-to-7-criteria. In patients with an ECOG (Eastern Cooperative Oncology Group) score of 0, EHD was detected in 11.2% compared to 18.2% in patients with an ECOG score of one or higher (*p* = 0.001). Furthermore, a significant positive correlation between elevated Child–Pugh scores and the presence of EHD was identified (*p* = 0.029).

The diagnostic performance of CT and MRI for the detection of EHD in reader group 1 and reader group 2 are presented in Table 2. The truth panel assessed if EHD was present or not.

CT detected significantly more EHD compared to MRI:

- Reader group 1: 105 lesions in CT vs. 35 lesions in MRI, odds ratio 0.29, CI: 0.2–0.44.
- Reader group 2: 98 lesions in CT vs. 53 lesions in MRI, odds ratio 0.48, CI: 0.34–0.67.

Furthermore, CT detected significantly more lymph node metastases and lung metastases compared to MRI in both reader groups (*p* < 0.001) as well as significantly more bone metastases in reader group 1 (*p* = 0.002). In reader group 2, the CT did not detect significantly more EHD than the MRI (*p* = 0.161).

Treatment recommendations by reader group 1 and reader group 2 are displayed in Table 3. Treatment recommendations were determined separately: at first, appropriate treatment was determined considering the intrahepatic tumor burden only.

**Figure 1:**
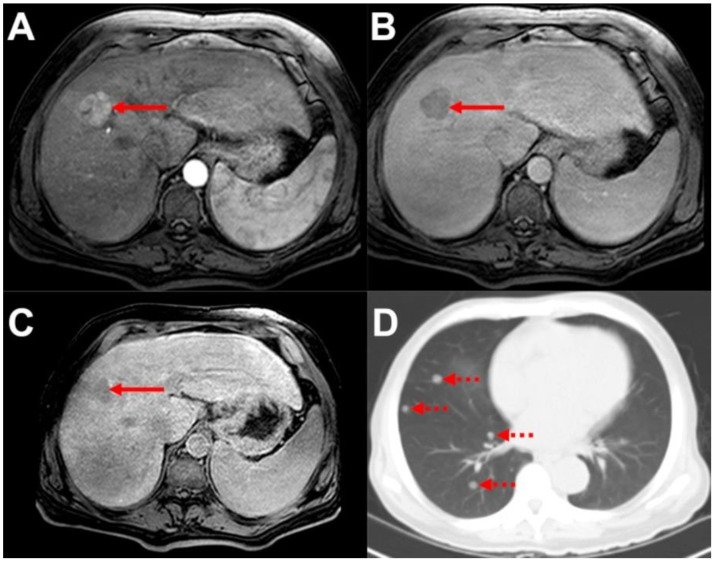
**EHD leading to change in management in a patient with solitary HCC.** (**A**): liver MRI, arterial phase and axial plane. (**B**): liver MRI, venous/transitional phase and axial plane. (**C**): liver MRI, hepatobiliary phase, and axial plane. (**D**): chest CT, lung window and axial plane. Note the solitary, typical HCC in liver segment VIII ((**A**–**C**), solid arrows) demonstrating arterial hypervascularization (**A**), venous/transitional phase washout (**B**), and hypointensity in the hepatobiliary phase ((**C**)—note is also made of visually reduced parenchymal contrast uptake due to impaired liver function). Based on hepatic disease, the treatment recommendation of RG1 was curative locoregional therapy. However, contrast-enhanced CT (**D**) revealed four pulmonary metastases in the right lung ((**D**), dashed arrows). The treatment recommendation was consequently changed to palliative systemic therapy.

In addition, treatment recommendations were determined considering the intrahepatic tumor burden as well as the prevalence of EHD. For the CT in reader group 1, EHD led to a change in management only in one patient (case demonstrated in Figure 1). In this patient, RG1 recommended curative locoregional therapy based on the MRI that revealed a solitary HCC. Due to several pulmonary metastases detected in additional CT staging, the recommendation was changed to palliative treatment.

**Figure 2:**
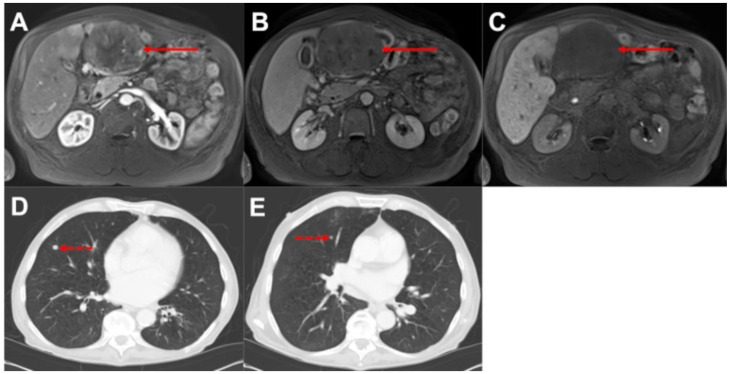
**No change in management despite CT-detected EHD.** (**A**): liver MRI, arterial phase, and axial plane. (**B**): liver MRI, venous/transitional phase, and axial plane. (**C**): liver MRI, hepatobiliary phase, and axial plane. (**D**,**E**): chest CT, lung window, and axial plane. Note the solitary, typical HCC in liver segment III ((**A**–**C**), solid arrows) demonstrating arterial hypervascularization (**A**), portalvenous phase washout (**B**), and hypointensity in the hepatobiliary phase (**C**). CT showed two additional pulmonary metastases in the right lung ((**D**,**E**), dashed arrows). However, EHD did not lead to a change of the therapy recommendation, as palliative treatment was already recommended due to close proximity of the primary tumor to central vascular structures.

**Figure 3:**
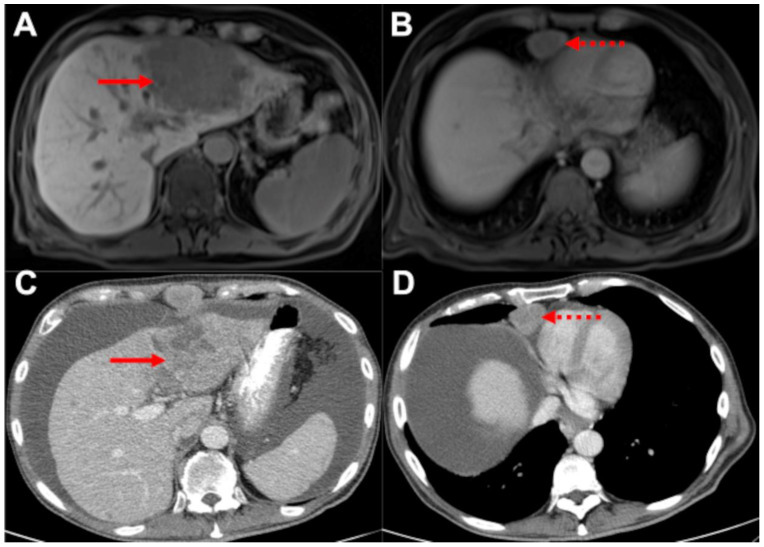
**No change in management as EHD is present in both MRI and CT.** (**A**,**B**): liver MRI, hepatobiliary phase, and axial plane. (**C**,**D**): abdomen and chest CT, soft tissue window, axial plane. Both MRI (**A**,**B**) and CT (**C**,**D**) show the HCC in liver segment II (solid arrows) and a cardiophrenic lymph node metastasis (dashed arrows). Therefore, palliative treatment was recommended, and additional CT staging did not result in a change in patient management. Paracentesis was performed between CT and MRI.

For the CT in reader group 2 as well as both reader groups for the MRI, no change in management based on EHD was noted. Figure 2 and Figure 3 show exemplary cases in which EHD did not lead to a change in management recommendation. In Figure 2, the CT revealed two additional pulmonary metastases that had not been detected by MRI. However, palliative treatment was already recommended due to the proximity of the solitary HCC to central vascular structures.

In Figure 3, CT did not lead to a change in management since a cardiophrenic lymph node metastasis was detected by both MRI and CT. Therefore, palliative systemic therapy was recommended by both reader groups.

## 4. Discussion

In this post-hoc analysis of the diagnostic study arm of the prospective phase II open-label, multicenter, randomized controlled SORAMIC trial, we investigated the performance of CT compared to hepatobiliary contrast liver MRI for the detection of EHD in HCC patients, and aimed to evaluate the impact of EHD on the most appropriate treatment recommendation.

CT detected significantly more EHD compared to liver MRI alone. CT detected significantly more lymph node metastases compared to MRI and detected significantly more lung metastases. The majority of lung metastases were not depicted by the MRI, due to a small lesion size and/or the scan range. However, further extrapulmonary metastases were present in more than half of the patients with lung metastases confirmed by the truth panel. Of note, MRI revealed more bone metastases compared to CT in our collective, even without fluid-sensitive sequences such as short tau inversion recovery (STIR). However, this observation did not achieve statistical significance. The most frequent site of EHD in our study was abdominal lymph nodes, followed by lung and bone. Previous publications have shown different results. In studies including 148, 151, and 342 HCC patients with EHD who underwent CT [19] or CT and MRI [9,20], respectively, the most frequent site of metastasis was the lung, followed by the abdominal lymph nodes and bone.

Our results show that more advanced local tumor stages, as indicated by the maximum lesion size, macrovascular invasion, and bilobar intrahepatic tumor spread, was associated with EHD. It was previously shown that EHD predominantly occurs in HCC patients with large primary intrahepatic tumors (TNM tumor stage T3 or T4) and blood vessel infiltration [19,21,22,23]. Considering these risk factors for the occurrence of EHD, it might be possible to reduce extensive imaging work-up in certain patients with advanced local tumor stages in HCC staging. This seems desirable since additional CT can lead to a higher cost, potential side effects by intravenous contrast agents, and increased logistical effort [24,25]. Our study showed that the detection of additional EHD in CT staging does not affect therapeutic decision-making in patients from the investigated population with advanced HCC, since these patients most often undergo palliative systemic therapy because of the advanced hepatic tumor load, macrovascular infiltration, and metastases already detected by MRI. In patients with macrovascular infiltration, there was no difference in treatment recommendation when evaluating the intrahepatic tumor load compared to evaluating the liver status and EHD in addition. However, further prospective studies are needed in order to identify patients in whom additional CT staging yields no impact on therapeutic decision-making, especially in patients with local disease in the early stages, qualifying for local therapy and in multifocal disease possibly qualifying for TACE.

Several authors have reported the superior diagnostic accuracy of dynamic gadoxetic-acid enhanced liver MRI for hepatic tumor staging compared to contrast-enhanced CT [13,26,27,28,29,30,31]. Within the diagnostic part of the SORAMIC trial, it was shown that gadoxetic-acid-enhanced hepatobiliary MRI allowed for more accurate decision-making in stratifying HCC patients to curative vs. palliative treatments compared to contrast-enhanced CT [17]. Whereas performing additional CT does not improve the diagnostic accuracy for local HCC staging, CT detected significantly more EHD compared to liver MRI in our study. However, the presence of EHD had no significant influence on the most appropriate recommendation, since hepatic disease extent already indicated palliative treatment rather than local/curative treatment (due to macrovascular invasion, bilobar involvement, or tumor size). Taking into consideration the high costs and limited availability of hepatobiliary contrast-enhanced MRI, investigating the added value of dedicated liver MRI with hepatocyte-specific contrast agents to CT in patients with advanced HCC would be an interesting objective for future studies.

It is known that the presence of EHD in HCC patients is associated with poor prognosis [9,21,32]. However, EHD does not aggravate the prognosis in all HCC patients. In 2016, a study including 214 HCC patients with and 719 patients without EHD detected lower survival rates at the presence of EHD only in patients with small intrahepatic HCC lesions [23]. The prognosis of patients with a larger-sized HCC was not significantly influenced by the presence of EHD. A potential explanation for this discrepancy may be that the prognosis of patients with advanced intrahepatic tumors is already poor and primarily defined by liver impairment due to tumor infiltration. Our results add to the literature as they demonstrate in a large-scale patient cohort that the impact of EHD on patient management is limited, especially in locally advanced tumors. However, the subset of patients in whom EHD influences the therapy decision needs to be defined in future studies in larger and more representative patient cohorts, including patients with a limited hepatic tumor load. These studies should assess if the risk factors for EHD identified in our study (large lesion size, macrovascular invasion, bilobar involvement, and an ECOG score of one or higher) can be reproduced and if they have an impact on therapy management. In addition, the impact of EHD on clinical outcome (i.e., progression-free and overall survival) should be investigated in future studies.

Several limitations of our study must be considered. First of all, palliative treatment was recommended for the majority of the included patients (424 out of 538). Thus, our results might have been biased by the selection criteria of the SORAMIC trial. Since the trial predominantly included patients with advanced HCC, the patients might have had an elevated risk of EHD and might have been more likely to be treated with a palliative approach. Therefore, our results might not be applicable for all HCC patients in general. Future studies with more balanced patient cohorts are needed to investigate if the selection bias in this study had a major impact on the change of treatment recommendation in HCC patients with EHD. However, local ablation was still recommended in a considerable number of patients (114 out of 538) in whom the presence of EHD might have changed the therapeutic approach. Furthermore, both CT reader groups detected more EHD than the truth panel. The truth panel analyzed all available CT and MRI image sets from baseline and each follow-up and had access to all clinical patient data. In contrast, the CT reader groups only analyzed the baseline CT images, which may have led to a bias due to the overdiagnosis of EHD. Due to the lack of follow-up examinations, suspicious lesions (e.g., adrenal masses representing lipid-poor adenomas) may have been misinterpreted as metastases more frequently by the CT readers compared to the truth panel. Moreover, not all pathological findings (e.g., lymph node metastases) were confirmed by a biopsy. Since histopathological evaluation was not always required for the clinical management of the individual patients of this cohort, a biopsy was not routinely performed. However, lesion detection and classification were performed by two independent, experienced reader groups to assure a high diagnostic accuracy. In addition, regarding the wider scan range, it is expected that whole-body CT identifies significantly more EHD compared to hepatobiliary liver MRI.

In conclusion, our study shows that EHD was detected significantly more frequently in patients with portal vein infiltration, macrovascular infiltration, bilobar liver involvement, and a higher maximum lesion diameter. CT staging detected significantly more EHD in HCC patients compared to dedicated liver MRI, but the presence of EHD had no significant impact on therapy decision in the analyzed cohort with a change in management in only one patient. Further prospective studies are needed in order to investigate if additional CT staging may be reduced in certain HCC patients (e.g., with locally advanced disease) and may help to individualize diagnostic imaging in the future with regard to patient comfort and resource management.

## Figures and Tables

**Table 1:** Baseline characteristics of all 538 included patients from the SORAMIC trial. The presence of EHD (EHDyes/EHDno) and respective *p*-values are displayed according to the decision of the truth panel.

		n	%	EHDyes	EHDno	*p*-Value
Sex (17 *)	Female	69	13.2	9	60	0.989
	male	452	86.8	63	389	
Age (y) (17 *)	Median			65	66	1
Race (38 *)	White	468	93.6	67	401	0.584
	Black	8	1.6	0	8	
	Asian	8	1.6	1	7	
	Other	16	3.2	3	13	
Previous HCC treatments (19 *)	TACE	97	51.9	11	86	0.549
	TAE	3	1.6	0	3	1
	Resection	41	21.9	3	38	0.313
	RFA	39	20.9	3	36	0.368
	Brachytherapy	7	1.3	2	5	0.553
Liver cirrhosis (23 *)	yes	418	81.2	51	367	0.081
	no	97	18.8	19	78	
ECOG (31 *)	0	375	74	42	333	**0.001**
	1	123	24.3	22	101	
	2	7	1.4	2	5	
	3	1	0.2	0	1	
	4	1	0.2	0	1	
HCC Diagnosis by (19 *)	Histology	223	43	36	187	0.414
	Imaging criteria	291	56.1	36	255	0.541
	Other	5	0.9	0	5	0.009
Cause of disease	Alcohol abuse	225	41.8	28	197	0.534
	Hepatitis B	57	10.6	7	50	0.89
	Hepatitis C	128	23.8	14	114	0.361
	NASH	49	9.1	6	43	0.091
	NAFLD	27	5	4	23	0.05
	Hemochromatosis	15	2.8	0	15	0.028
	Cryptogenic	50	9.3	13	37	0.093
	other	6	1.1	1	5	
Child–Pugh Score (23 *)	A	458	88.9	62	396	0.323
	B	55	10.7	8	47	
	C	2	0.4	1	1	
Child–Pugh Points (24 *)	4	2		0	2	**0.029**
	5	330	64.2	39	291	
	6	127	24.7	25	101	
	7	47	9.1	3	44	
	8	6	1.2	2	4	
	10	2	0.4	1	1	
BCLC stage (25 *)	0	6	1.2	1	5	**<0.001**
	A	93	18.1	2	91	
	B	144	28.1	19	125	
	C	269	52.4	48	221	
	D	1	0.2	1	0	
PVI	y	174	32.3	37	137	**<0.001**
	n	364	67.8	37	327	
MVI	y	191	35.5	42	149	**<0.001**
	n	347	64.4	32	315	
Disease spread (21 *)	Unilobar	222		20	202	**0.006**
	Bilobar	295		53	243	
Hypervascular lesions	0–4	354		44	310	0.269
	>4	184		30	154	
Maximum lesion diameter (cm)	Median	5.9		8.2	5.8	**0.002**
	IQR	3.6–9.9		5.3–10.5	3.4–9.8	
Up-to-7-criteria (39 *)	Within up-to-7	116		2	114	**<0.001**
	Out of up-to-7	383		67	316	

* n/a. CT RG1. BCLC, Barcelona Clinic Liver Cancer; ECOG, Eastern Cooperative Oncology Group; HCC, hepatocellular carcinoma; IQR, inter-quartile range; MVI, Macrovascular infiltration; NAFLD, non-alcoholic fatty liver disease; NASH, non-alcoholic steatohepatitis; PVI, portal vein infiltration; RFA, radio-frequency ablation; TACE, transarterial chemoembolization; and TAE, transarterial embolization. *p*-values < 0.05 are highlighted in bold font.

**Table 2:** Diagnostic performance of CT and MRI for the detection of EHD (RG1 and RG2).

EHD Location	Reader Group	Imaging Modality	EHDno	EHDyes	*p*-Value
Total	RG1	CT	433	105	*p* < 0.001
		MRI	503	35	
	RG2	CT	440	98	*p* < 0.001
		MRI	485	53	
	Truth panel	CT and MRI	464	74	
Lymph nodes	RG1	CT	443	95	*p* < 0.001
		MRI	504	34	
	RG2	CT	459	79	*p* < 0.001
		MRI	494	44	
Lung	RG1	CT	522	16	*p* < 0.001
		MRI	538	0	
	RG2	CT	526	12	*p* = 0.019
		MRI	535	3	
Bone	RG1	CT	537	1	*p* = 0.002
		MRI	536	2	
	RG2	CT	534	4	*p* = 0.161
		MRI	529	9	
Other	RG1	CT	538	0	*p* = 1
		MRI	538	0	
	RG2	CT	525	13	*p* < 0.001
		MRI	538	0	

CT, computed tomography; EHD, extrahepatic disease; MRI, magnetic resonance imaging; RG1, reader group 1; and RG2, reader group 2.

**Table 3:** Most appropriate therapy recommendation by RG1 and RG2 based on hepatic disease only and both hepatic disease and EHD. The therapy recommendation was compared to the recommendation by the truth panel (based on hepatic disease and EHD in all imaging modalities, respectively).

	Reader Group		Therapy Recommendation vs. Truth Panel	
			Agreement (%)	IQR (%)
**Liver only**	RG1	CT	80.9	77.3–84.1
		MRI	81.8	78.3–85.0
	RG2	CT	78.1	74.3–81.5
		MRI	80.3	76.7–83.6
**Liver + EHD**	RG1	CT	80.7	77.1–83.9
		MRI	81.8	78.3–85.0
	RG2	CT	77.7	73.9–81.1
		MRI	80.3	76.7–83.6

CT, computed tomography; EHD, extrahepatic disease; IQR, inter-quartile range; MRI, magnetic resonance imaging; RG1, reader group 1; and RG2, reader group 2.

## Data Availability

Not applicable.
